# Association between relative fat mass and risk of arthritis: a study based on populations in China and the United States

**DOI:** 10.3389/fnut.2025.1555135

**Published:** 2025-09-02

**Authors:** Yang Yang, Yuanfan Li, Ruixing Shui, Dapeng Li

**Affiliations:** Department of Spinal Surgery, Affiliated Hospital of Jiangsu University, Zhenjiang, China

**Keywords:** RFM, arthritis, NHANES, CHARLS, obesity

## Abstract

This study examined the association between relative fat mass (RFM) and the prevalence of arthritis in two distinct populations: one from China and the other from the United States. The findings indicated a non-linear relationship between RFM and the development of arthritis. A robust positive correlation was identified in the US male population, while no such correlation was observed in the Chinese male population. In the American female population, a non-linear correlation was observed between RFM and arthritis, with elevated RFM below the threshold of 35.85 exhibiting a modest decrease in the risk of arthritis, and elevated RFM above the threshold demonstrating a substantial increase in the risk of arthritis. A similar trend was observed in Chinese women; however, the protective effect was not significant below the threshold (*p* > 0.05). Subgroup analyses further revealed that factors such as hypertension and smoking significantly altered the association between RFM and arthritis in the US population of both genders, whereas the relationship between RFM and arthritis was relatively stable in the Chinese female population. The present study suggests that increased RFM is associated with the prevalence of arthritis in men, and that maintaining optimal levels of RFM may reduce the risk of arthritis in women. RFM, as a new independent arthritis risk factor, can be used for screening and long-term monitoring of patients with arthritis, as well as to assess the effectiveness of various treatment modalities.

## 1 Introduction

Arthritis is a prevalent ailment defined by impaired joint mobility and discomfort. The two predominant forms are osteoarthritis (OA) and rheumatoid arthritis (RA). OA is a chronic degenerative disease that mainly involves the gradual degeneration and inflammation of articular cartilage that bears the weight of the body. RA is a systemic autoimmune disorder marked by persistent synovial inflammation, which can culminate in cartilage and bone destruction. As the global population ages and obesity rates rise, the incidence of arthritis is increasing and has become a major public health problem (1).

Obesity is widely regarded as a significant risk factor contributing to the onset of arthritis (2–5). Conventional methods for evaluating obesity are limited in their capacity to differentiate between body fat and muscle mass (6). Consequently, there is a necessity for the development of more scientific measurement indicators.

In recent years, novel obesity assessment indicators such as the visceral fat index (VAI) (7, 8), the weight-adjusted waist index (WWI) (9, 10), and relative fat mass (RFM) (11, 12) have been proposed in succession, in an attempt to more accurately reflect the impact of fat distribution on health. Among these, RFM is a recently emergent indicator that has garnered increasing attention. RFM has been shown to be more precise than the body mass index (BMI) in estimating the percentage of body fat in adults, as verified by dual-energy X-ray absorptiometry (11). Additionally, studies have identified a significant association between RFM and various health conditions, including periodontitis (13), diabetes (14), and cardiovascular disease (12).

Notwithstanding the investigation of the correlation between RFM and a variety of diseases in preceding studies, the link to arthritis remains ambiguous. Consequently, this research set out to utilize data from the (National Health and Nutrition Examination Survey) NHANES and (China Health and Retirement Longitudinal Study) CHARLS to further investigate the association between RFM and arthritis, thereby offering novel insights into research in this domain.

## 2 Methodologies

### 2.1 Study population

**Figure 1:**
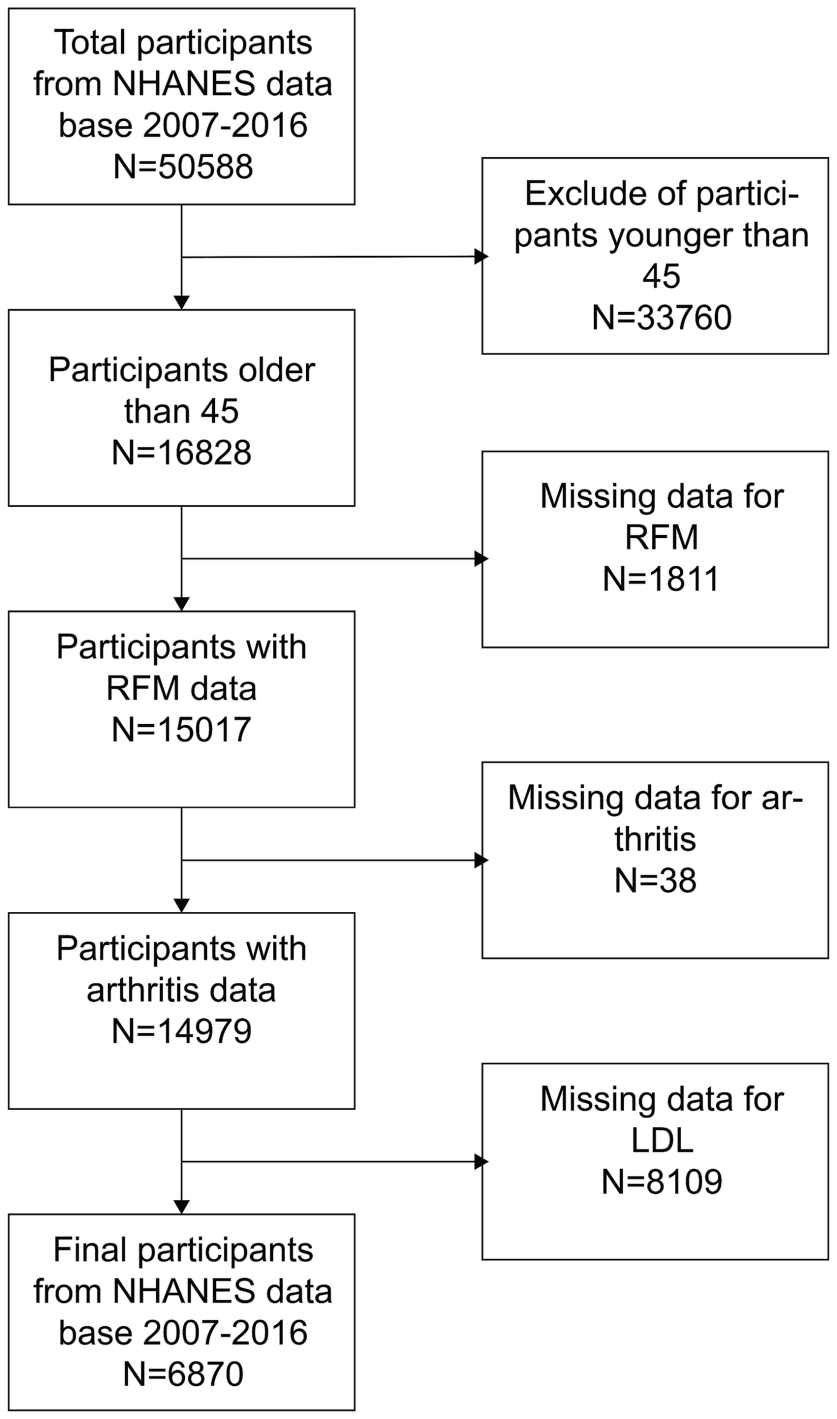
U.S. patient choice flowchart.

**Figure 2:**
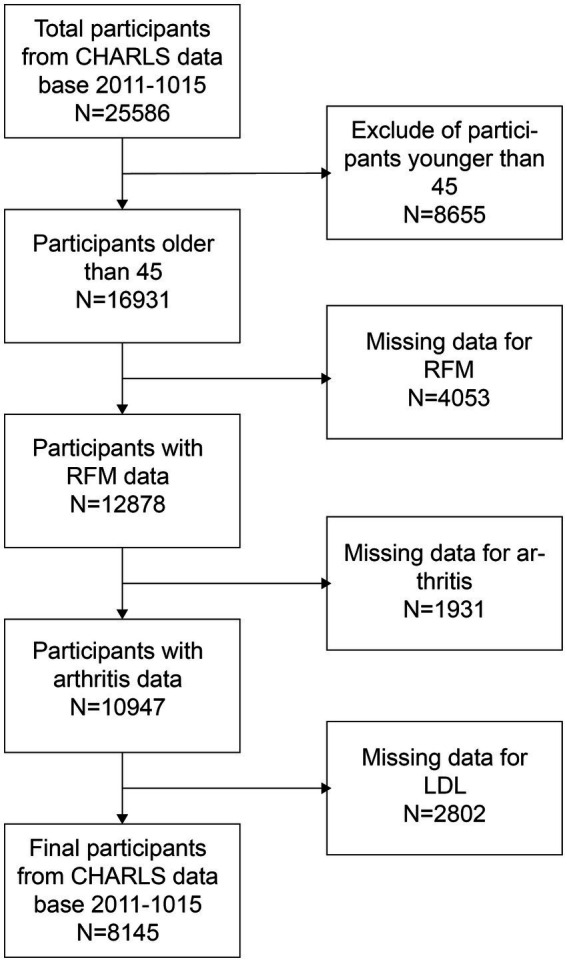
Chinese patient choice flowchart.

The NHANES is a comprehensive health and nutrition assessment survey conducted biennially. The Research Ethics Review Committee of the National Center for Health Statistics reviews and approves the survey. The official website contains all relevant data. The current analysis relies on NHANES data gathered from 2007 to 2016, which included a total of 50,588 participants. Additionally, data from 25,586 participants in the 2011 CHARLS database, along with their follow-up data from 2015, were also collected. The CHARLS is a large-scale research database aimed to gather data on the health, economy, society, and family of China’s middle-aged and elderly population (45 years old and above). It is a valuable resource for research on the health status, quality of life, and social security of the elderly in China, as well as a substantial data foundation for policy formulation and academic research. To ensure the integrity of the research, individuals younger than 45 years old and lacking data on RFM, arthritis, and LDL-C were excluded. As illustrated in Figures 1, 2, 6,870 and 8,145 participants were included in the survey, respectively.

### 2.2 Calculation of RFM

RFM is calculated using the following formula: 64 − (20 × height (cm)/waist circumference (cm)) + (12 × sex), where sex is assigned a value of 1 for females and 0 for males (11). During the CHARLS and NHANES surveys, experts measured the subjects’ waists circumference and heights.

### 2.3 Diagnosis of arthritis

Arthritis status was based on self-reported physician diagnosis. NHANES participants were asked if they had ever been diagnosed with arthritis by a doctor, while CHARLS participants were asked whether they had been diagnosed with arthritis or rheumatism.

### 2.4 Covariate data collection

This study extracted a multitude of potential covariates from the extant literature that may exert an influence on the relationship between RFM and arthritis. The analysis included the lifestyle behavioral factors: alcohol use, smoking status, and sleep duration. Chronic disease status: hypertension, heart disease, stroke, and diabetes. In the context of NHANES, “heart disease” refers to a self-reported response indicating the presence of coronary heart disease, angina pectoris, congestive heart failure, or myocardial infarction. In CHARLS, “heart problems” refers to a medical diagnosis. Laboratory indicators include high-density lipoprotein cholesterol (HDL-C) and low-density lipoprotein cholesterol (LDL-C).

In this paper, the covariate “race” was considered mainly in the NHANES sample in the U.S. because of its diverse racial composition, and was not included in the Chinese population group because of the lack of race-related data in the CHARLS database.

### 2.5 Statistical analysis

The following statistical analyses were performed in this study. Standard deviations and means were used for continuous variables, categorical variables were measured using percentages. Multiple imputation was used for missing data. For continuous variables, weighted Student’s t-tests were used, and for categorical variables, weighted chi-square tests were utilized. The purpose of these tests was to evaluate the differences between the groups in RFM tertiles. A multivariate logistic regression model was used to examine the link between RFM and arthritis. A cross-sectional study was conducted on the NHANES data, and a longitudinal study was conducted on the CHARLS data. Model 1 did not adjust for any confounders. In Model 2, race and age have been taken into account, and Model 3 adjusted for all covariates. Curve fitting and threshold analysis were used to assess the relationship between RFM and arthritis. Finally, potential differences in the relationship between the two were explored among different populations using subgroup analyses and interaction tests. EmpowerStats software was used for each statistical assessment. Statistical significance was established at *p* < 0.05.

## 3 Results

### 3.1 Basic characteristics of participants in the two countries

Table 1 presents a comparison of the characteristics of the study populations in China and the United States. The RFM of American men (29.92) was found to be significantly higher than that of Chinese men (24.95). However, there was minimal discrepancy in HDL-C and LDL-C levels between the two groups, and sleep duration exhibited a high degree of similarity as well. Furthermore, the proportion of highly educated American men was significantly higher than that of Chinese men. With respect to marital status, 72.35% of American men were either married or had a partner, which was lower than the 91.19% of Chinese men. With regard to smoking rates, Chinese men exhibit a significantly higher prevalence than their American counterparts. Conversely, American men demonstrate a higher percentage of alcohol consumption. Hypertension, diabetes, heart disease, and stroke exhibit a higher prevalence among American men than among Chinese men. The prevalence of arthritis was 43.15% among Chinese men, which is higher than the 33.92% observed among American men.

**Table 1:** Baseline table of population groups in the United States and China.

Gender	NHANES		*p*-value	CHARLS		*p*-value
	Male	Female		Male	Female	
*N*	3,331	3,539		3,780	4,365	
Age	62.11 ± 10.62	62.04 ± 10.82	0.806	59.62 ± 8.93	58.39 ± 9.16	<0.001
RFM	29.92 ± 4.58	43.22 ± 5.04	<0.001	24.95 ± 4.25	39.91 ± 4.30	<0.001
HDL-C	50.16 ± 15.02	60.07 ± 16.47	<0.001	50.82 ± 16.13	51.67 ± 14.41	0.012
LDL-C	112.71 ± 36.55	119.74 ± 36.21	<0.001	112.25 ± 33.74	120.62 ± 35.30	<0.001
Sleeping time	6.97 ± 1.51	7.03 ± 1.53	0.116	6.46 ± 1.79	6.23 ± 1.94	<0.001
Race			0.095	/	/	/
Mexican American	427 (12.82%)	497 (14.04%)		/	/	/
Other Hispanic	370 (11.11%)	445 (12.57%)		/	/	/
Non-Hispanic White	1,567 (47.04%)	1,588 (44.87%)		/	/	/
Non-Hispanic Black	653 (19.60%)	702 (19.84%)		/	/	/
Other Race - Including Multi-Racial	314 (9.43%)	307 (8.67%)		/	/	/
Education level			0.588			<0.001
Less than high school	966 (29.00%)	1,011 (28.57%)		3,276 (86.67%)	4,104 (94.02%)	
High school diploma (including GED)	739 (22.19%)	822 (23.23%)		446 (11.80%)	243 (5.57%)	
More than high school	1,626 (48.81%)	1706 (48.21%)		58 (1.53%)	18 (0.41%)	
Marital status			<0.001			<0.001
Married/Living with Partner	2,410 (72.35%)	1864 (52.67%)		3,447 (91.19%)	3,718 (85.18%)	
Never Married/Divorced/Other	921 (27.65%)	1,675 (47.33%)		333 (8.81%)	647 (14.82%)	
Drinking			<0.001			<0.001
No	578 (17.35%)	1,525 (43.09%)		2,278 (60.26%)	4,164 (95.40%)	
Yes	2,753 (82.65%)	2014 (56.91%)		1,502 (39.74%)	201 (4.60%)	
Hypertension			0.002			<0.001
No	1,681 (50.47%)	1,655 (46.76%)		2,870 (75.93%)	3,144 (72.03%)	
Yes	1,650 (49.53%)	1884 (53.24%)		910 (24.07%)	1,221 (27.97%)	
Diabetes			0.019			0.004
No	2,663 (79.95%)	2,908 (82.17%)		3,581 (94.74%)	4,068 (93.20%)	
Yes	668 (20.05%)	631 (17.83%)		199 (5.26%)	297 (6.80%)	
Arthritis			<0.001			<0.001
No	2,201 (66.08%)	1897 (53.60%)		2,149 (56.85%)	2045 (46.85%)	
Yes	1,130 (33.92%)	1,642 (46.40%)		1,631 (43.15%)	2,320 (53.15%)	
Heart diseases			<0.001			<0.001
No	2,787 (83.67%)	3,160 (89.29%)		3,381 (89.44%)	3,800 (87.06%)	
Yes	544 (16.33%)	379 (10.71%)		399 (10.56%)	565 (12.94%)	
Stroke			0.773			0.217
No	3,149 (94.54%)	3,340 (94.38%)		3,681 (97.38%)	4,269 (97.80%)	
Yes	182 (5.46%)	199 (5.62%)		99 (2.62%)	96 (2.20%)	
Smoke			<0.001			<0.001
No	1,301 (39.06%)	2,167 (61.23%)		936 (24.76%)	4,036 (92.46%)	
Yes	2030 (60.94%)	1,372 (38.77%)		2,844 (75.24%)	329 (7.54%)	

Data are expressed as Mean + SD/*N* (%). HDL-C, High-density lipoprotein cholesterol; LDL-C, Low-density lipoprotein cholesterol; RFM, relative fat mass.

The RFM of American women (43.22) surpassed that of Chinese women (39.91). However, the HDL-C and sleep duration of the former exceeded those of the latter. The proportion of highly educated American women was significantly higher than that of Chinese women, and the proportion of married or partnered women was 52.67%, which was lower than that of Chinese women (85.18%). With respect to lifestyle factors, American women exhibit smoking and drinking rates that are considerably higher than those observed among Chinese women. Hypertension, diabetes, heart disease, and stroke are conditions that are more prevalent among American women than Chinese women. The prevalence of arthritis among Chinese women (53.15%) exceeds that among American women (46.40%).

### 3.2 Relationship between RFM and arthritis

In Table 2, the association between RFM and arthritis in the US and Chinese male and female populations is further analyzed. When RFM was employed as a continuous variable, a significant positive association between RFM and arthritis in U.S. men was observed (Model 3: OR = 1.05, 95%CI: 1.03–1.08). Conversely, this significant association was not observed in Chinese men (Model 3: OR = 1.01, 95%CI: 0.99–1.02). The positive association between RFM and arthritis proved to be equally significant for women in the United States (Model 3: OR = 1.07, 95% CI: 1.05–1.09). A similar outcome was observed in Chinese women, who also demonstrated a statistically significant positive association (Model 3: OR = 1.03, 95% CI: 1.02–1.05). However, the strength of the correlation was found to be lower in Chinese women compared to their U.S. counterparts.

**Table 2:** The association between RFM and arthritis.

	Model 1	Model 2	Model 3
RFM (continuous)			
NHANES			
RFM (male)	1.08 (1.06, 1.10)	1.07 (1.05, 1.09)	1.05 (1.03, 1.08)
RFM (female)	1.08 (1.06, 1.09)	1.08 (1.06, 1.09)	1.07 (1.05, 1.09)
CHARLS			
RFM (male)	1.00 (0.99, 1.02)	1.00 (0.99, 1.02)	1.01 (0.99, 1.02)
RFM (female)	1.03 (1.02, 1.04)	1.02 (1.01, 1.04)	1.03 (1.02, 1.05)
RFM Tertile			
NHANES			
RFM (male)			
Q1 (11.93–28.28)	1	1	1
Q2 (28.28–31.87)	1.27 (1.06, 1.53)	1.18 (0.98, 1.43)	1.09 (0.89, 1.33)
Q3 (31.87–43.56)	2.22 (1.86, 2.65)	1.93 (1.60, 2.33)	1.65 (1.34, 2.03)
P for trend	<0.0001	<0.0001	<0.0001
RFM (female)			
Q4 (23.62–41.34)	1	1	1
Q5 (41.34–45.71)	1.39 (1.18, 1.64)	1.37 (1.15, 1.63)	1.34 (1.11, 1.61)
Q6 (45.71–56.21)	2.26 (1.92, 2.67)	2.31 (1.94, 2.75)	1.98 (1.63, 2.41)
P for trend	<0.0001	<0.0001	<0.0001
CHARLS			
RFM (male)			
Q7 (7.79–23.17)	1	1	1
Q8 (23.17–26.96)	1.00 (0.85, 1.17)	0.99 (0.85, 1.16)	1.01 (0.86, 1.18)
Q9 (26.96–37.61)	1.03 (0.88, 1.21)	1.03 (0.88, 1.20)	1.07 (0.90, 1.27)
P for trend	0.7174	0.7533	0.4919
RFM (female)			
Q10 (23.49–38.22)	1	1	1
Q11 (38.22–42.14)	1.14 (0.99, 1.32)	1.14 (0.98, 1.31)	1.19 (1.02, 1.38)
Q12 (42.14–52.94)	1.33 (1.15, 1.54)	1.26 (1.09, 1.46)	1.40 (1.16, 1.60)
P for trend	0.0001	0.0022	0.0002

Data are expressed as OR (95%CI) *p* value. Model 1 adjusts for: None. Model 2 adjusts for: race, age. Model 3 adjusts for: age, race, education level, marital status, drinking and smoking habits, diabetes, hypertension, heart disease, stroke, sleep duration, HDL-c, and LDL-c.

When RFM was utilized as a grouping variable, the risk of arthritis in US men increased significantly with increasing RFM tertiles. In model 3, the risk of developing arthritis in the high RFM group was 1.65 times higher than that in the low RFM group (OR = 1.65, 95%CI: 1.34–2.03) in US men, with a significant convergence (P for trend<0.0001). Conversely, no substantial trend of elevated arthritis risk was observed among Chinese men across RFM subgroups (P for trend = 0.4919). The present study found that, among women, the risk of arthritis in the high RFM group was 1.98 times higher than that in the low RFM group (OR = 1.98, 95%CI: 1.63–2.41). Similarly, the risk in the high RFM group was 1.40 times higher in Chinese women (OR = 1.40, 95%CI: 1.16–1.60). These findings indicate an elevated risk, albeit at a lower magnitude than observed in US women.

**Figure 3:**
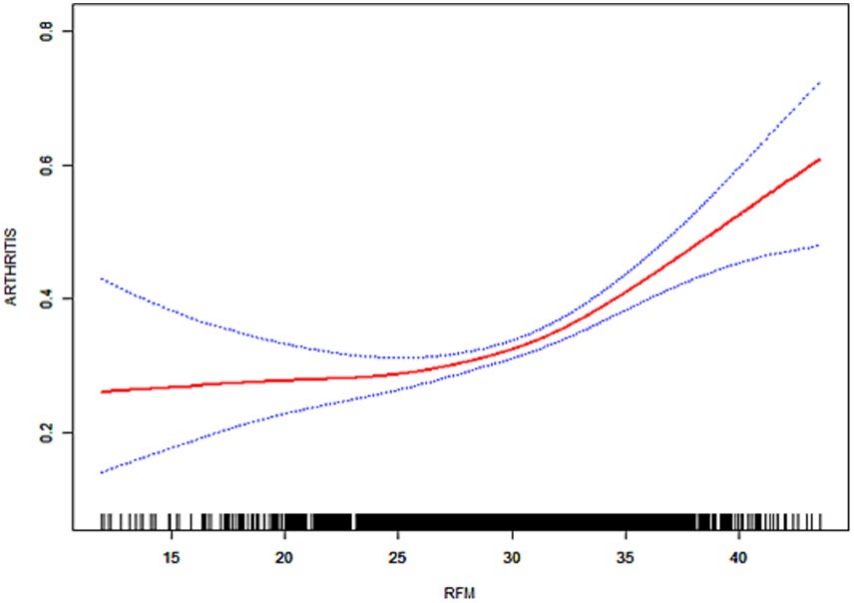
Smoothed curve fitting for the U.S. population (male).

**Figure 4:**
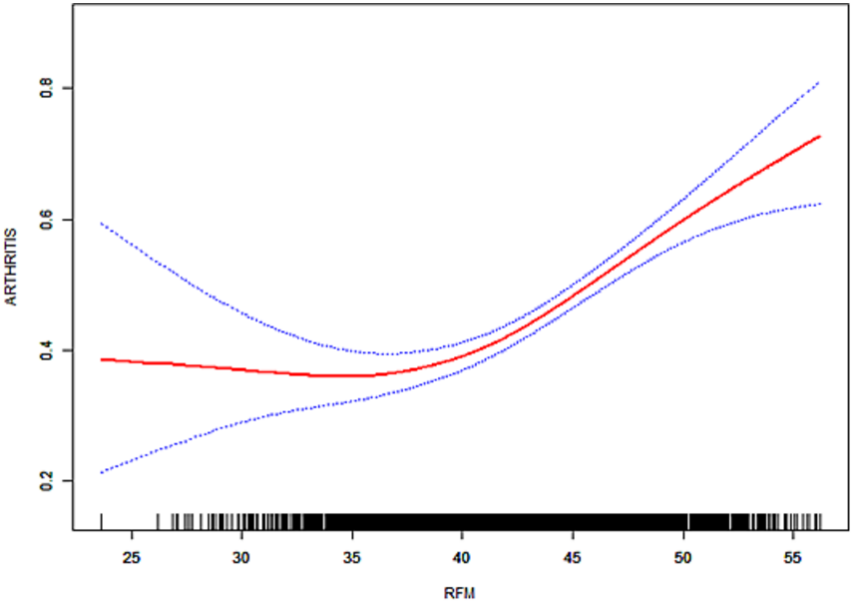
Smoothed curve fitting for the U.S. population (female).

**Figure 5:**
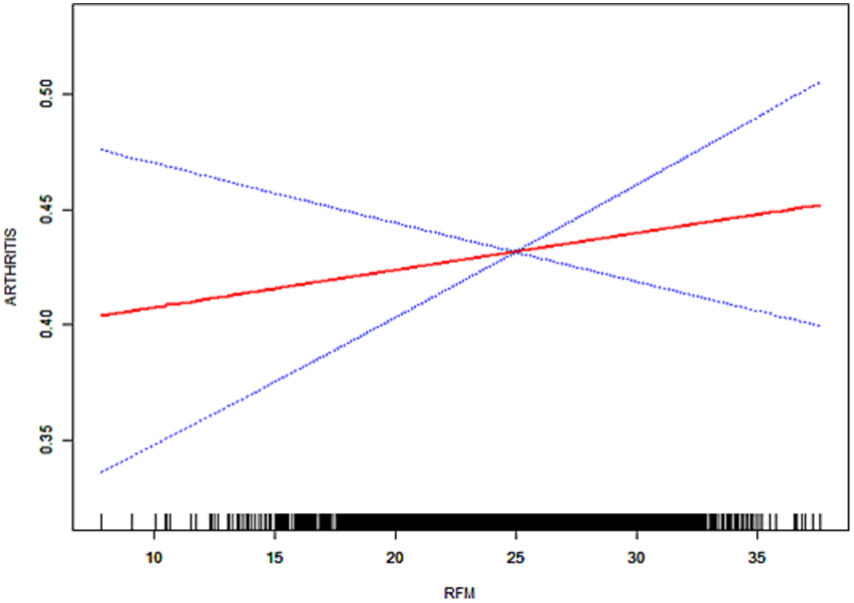
Smoothed curve fitting for the Chinese population (male).

**Figure 6:**
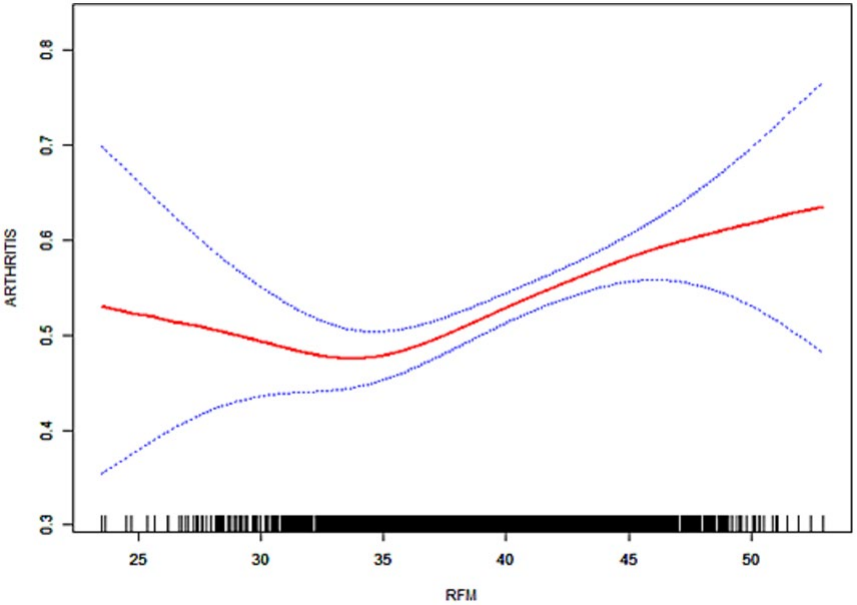
Smoothed curve fitting for the Chinese population (female).

As illustrated in Figures 3–6, the implementation of smoothed curves revealed a positive correlation between RFM and arthritis in all populations except Chinese men. Subsequent threshold analysis was conducted (Table 3). For the male subjects in the US group, the RFM threshold was determined to be 31.44. The presence of RFM below the established threshold did not demonstrate a substantial correlation with the risk of arthritis [odds ratio (OR) = 1.02, *p* = 0.3020]. However, above the threshold, a significant increase in risk was observed, with a 12% increase in the likelihood of developing the disease for each additional unit (OR = 1.12, *p* < 0.0001). In contrast, the RFM threshold for Chinese men was 27.11, and the association between RFM and arthritis risk did not reach statistical significance either below or above the threshold (*p* > 0.05).

**Table 3:** Threshold analysis.

Outcome	NHANES (male)	NHANES (female)	CHARLS (male)	CHARLS (female)
Inflection point (K)	31.44	35.85	27.11	34.13
RFM < K slope	1.02 (0.99, 1.05) 0.3020	0.92 (0.85, 0.99) 0.0306	1.02 (0.99, 1.04) 0.1882	0.94 (0.87, 1.01) 0.0701
RFM > K slope	1.12 (1.08, 1.17) < 0.0001	1.09 (1.07, 1.11) < 0.0001	0.98 (0.94, 1.03) 0.4407	1.05 (1.03, 1.07) < 0.0001
Log-likelihood ratio test	<0.001	<0.001	0.266	0.005

Data are expressed as OR (95%CI) *p* value. Adjust for: gender, age, race, education level, marital status, drinking and smoking habits, diabetes, hypertension, heart disease, stroke, sleep duration, HDL-c, and LDL-c.

The RFM threshold was determined to be 35.85 for American women. Below this value, RFM demonstrated a negative correlation with arthritis (OR = 0.92, *p* = 0.0306). Conversely, above the threshold, a significant increase in the risk of developing the disease was observed with higher RFM, with a 9% elevated risk per unit increase (OR = 1.09, *p* < 0.0001). The RFM threshold for Chinese women was determined to be 34.13, and no significant association was observed below this threshold (OR = 0.94, *p* = 0.0701). However, above the threshold, there was a 5% increase in risk (OR = 1.05, *p* < 0.0001). In the log-likelihood ratio test, the model fit was significantly improved with the introduction of the threshold in both the female and US male groups. However, no significant improvement was seen in the Chinese male group.

### 3.3 Subgroup analyses

As demonstrated in Tables 4, 5, subgroup analyses were used to determine how population stratification factors affected RFM and arthritis. For U.S. men, statistically significant differences were identified among subgroups based on age (*p* = 0.0225), education level (*p* = 0.0321), hypertension (*p* = 0.0386), smoking (*p* = 0.0133), and stroke (*p* = 0.0002). For U.S. women, the association between RFM and arthritis differed significantly between subgroups of race (*p* = 0.0032), hypertension (*p* = 0.01), diabetes (*p* = 0.0223), smoking (*p* = 0.0008), and alcohol consumption (*p* = 0.0231). In the Chinese female population, the *p* values of the tests for the subgroup interactions were all high, indicating that the effect of RFM on arthritis risk varied relatively little between subgroups of the population.

**Table 4:** Subgroup analysis of the U.S. population.

	NHANES (male)		NHANES (female)	
	OR (95%CI)	P for interaction	OR (95%CI)	P for interaction
Age		0.0225		0.4911
<=65	1.07 (1.05, 1.10)		1.07 (1.05, 1.09)	
>65	1.03 (1.00, 1.06)		1.06 (1.03, 1.09)	
Race		0.2069		0.0032
Mexican American	1.10 (1.03, 1.18)		1.03 (0.98, 1.09)	
Other Hispanic	1.14 (1.06, 1.22)		1.10 (1.04, 1.16)	
Non-Hispanic White	1.05 (1.02, 1.08)		1.04 (1.02, 1.07)	
Non-Hispanic Black	1.05 (1.01, 1.09)		1.11 (1.07, 1.15)	
Other race	1.06 (0.98, 1.16)		1.14 (1.07, 1.22)	
Marital status		0.2218		0.1488
Never married/divorced	1.04 (1.01, 1.08)		1.08 (1.05, 1.10)	
Married/living with partner	1.07 (1.04, 1.09)		1.06 (1.03, 1.08)	
Education level		0.0321		0.5277
Less than high school	1.03 (0.99, 1.06)		1.06 (1.03, 1.09)	
High school diploma	1.05 (1.01, 1.09)		1.06 (1.02, 1.09)	
More than high school	1.09 (1.06, 1.12)		1.08 (1.05, 1.10)	
Hypertension		0.0386		0.01
No	1.08 (1.05, 1.11)		1.05 (1.02, 1.07)	
Yes	1.04 (1.01, 1.07)		1.09 (1.06, 1.11)	
Diabetes		0.0598		0.0223
No	1.05 (1.03, 1.07)		1.06 (1.04, 1.08)	
Yes	1.10 (1.05, 1.14)		1.12 (1.07, 1.16)	
Smoke		0.0133		0.0008
No	1.09 (1.06, 1.13)		1.09 (1.07, 1.12)	
Yes	1.04 (1.02, 1.07)		1.04 (1.01, 1.06)	
Drinking		0.6624		0.0231
No	1.07 (1.02, 1.12)		1.09 (1.06, 1.12)	
Yes	1.06 (1.03, 1.08)		1.05 (1.03, 1.07)	
Heart diseases		0.6424		0.6989
No	1.06 (1.03, 1.08)		1.07 (1.05, 1.09)	
Yes	1.07 (1.02, 1.12)		1.06 (1.01, 1.11)	
Stroke		0.0002		0.6339
No	1.07 (1.04, 1.09)		1.07 (1.05, 1.09)	
Yes	0.92 (0.86, 1.00)		1.05 (0.98, 1.12)	
Sleeping time		0.6457		0.7787
<=7	1.07 (1.04, 1.09)		1.06 (1.04, 1.08)	
7 ~ 9	1.05 (1.02, 1.09)		1.07 (1.04, 1.10)	
>9	1.03 (0.95, 1.12)		1.09 (1.02, 1.16)	

Data are expressed as OR (95%CI) *p* value.

**Table 5:** Subgroup analysis of the Chinese population.

	CHARLS (male)		CHARLS (female)	
	OR (95%CI)	P for interaction	OR (95%CI)	P for interaction
Age		0.0276		0.4669
<=65	1.00 (0.98, 1.01)		1.03 (1.01, 1.05)	
>65	1.04 (1.00, 1.07)		1.04 (1.01, 1.07)	
Marital status		0.6614		0.0906
Never Married/divorced	0.99 (0.94, 1.05)		1.00 (0.97, 1.04)	
Married/living with partner	1.01 (0.99, 1.02)		1.04 (1.02, 1.06)	
Education level		0.2788		0.5735
Less than high school	1.01 (0.99, 1.02)		1.03 (1.02, 1.05)	
High school diploma	0.99 (0.94, 1.04)		1.05 (0.97, 1.13)	
More than high school	1.23 (0.92, 1.66)		1.68 (0.55, 5.16)	
Hypertension		0.1654		0.0753
No	1.00 (0.98, 1.02)		1.04 (1.02, 1.06)	
Yes	1.03 (0.99, 1.06)		1.01 (0.98, 1.04)	
Diabetes		0.52		0.6651
No	1.01 (0.99, 1.02)		1.03 (1.02, 1.05)	
Yes	0.98 (0.91, 1.06)		1.02 (0.95, 1.09)	
Smoke		0.6948		0.5089
No	1.00 (0.97, 1.03)		1.03 (1.02, 1.05)	
Yes	1.01 (0.99, 1.03)		1.05 (1.00, 1.11)	
Drinking		0.2024		0.268
No	1.01 (0.99, 1.03)		1.03 (1.02, 1.05)	
Yes	0.99 (0.96, 1.02)		1.07 (1.00, 1.14)	
Heart diseases		0.859		0.9734
No	1.01 (0.99, 1.02)		1.03 (1.02, 1.05)	
Yes	1.00 (0.95, 1.05)		1.03 (0.99, 1.08)	
Stroke		0.3496		0.292
No	1.00 (0.99, 1.02)		1.03 (1.02, 1.05)	
Yes	1.06 (0.95, 1.18)		1.10 (0.98, 1.23)	
Sleeping time		0.9039		0.5726
<=7	1.01 (0.99, 1.03)		1.03 (1.02, 1.05)	
7 ~ 9	1.00 (0.97, 1.04)		1.03 (1.00, 1.06)	
>9	0.99 (0.91, 1.08)		1.08 (0.99, 1.17)	

Data are expressed as OR (95%CI) *p* value.

In summary, the relationship between RFM and arthritis is strongly influenced by multiple demographic and health challenge factors in both male and female populations in the United States. In contrast, in Chinese women, this effect was more homogeneous, with no significant interactions between subgroups.

## 4 Discussion

The present study further reveals that the correlation between RFM and arthritis exhibits significant differences across gender and country populations. In the American male population, RFM demonstrated a positive association with the risk of arthritis, exhibiting a significant non-linear relationship. Conversely, no substantial correlation with arthritis risk was identified in Chinese men, irrespective of RFM, suggesting a minimal contribution of RFM in this demographic. This phenomenon may be attributed to the relatively low RFM values observed in the male population, particularly in Chinese men, and the overall low prevalence of obesity in this demographic. The limited variability in RFM among Chinese men over the age of 45 years old further complicates the ability of RFM to accurately reflect variations in arthritis risk.

In the female population, the threshold for RFM in American women is 35.85, below which RFM may even be slightly protective, but above which the risk of arthritis increases significantly with elevated RFM. In contrast, the threshold for RFM in Chinese women was 34.13, and only when this threshold was exceeded did the risk of arthritis increase significantly, with a lower intensity than in US women. When the values fell below this threshold, the analysis indicated a possible protective effect in Chinese women, although the *p* value was not significant (*p* > 0.05). A number of studies have been conducted that lend support to the hypothesis that inflammation resulting from excessive adiposity, as well as metabolic disturbances associated with insufficient adiposity, both contribute to an increased risk of arthritis (15, 16).

Recent research has indicated a significant association between RFM and various health problems. Xiao et al. (17) discovered a non-linear relationship between RFM and diabetes risk in a study of Japanese adults. The RFM threshold for women is 39.23, and the risk of diabetes increases significantly when this value is exceeded (HR: 1.39). In the male population, RFM levels exceeding 23.08 were found to be associated with an elevated risk of diabetes (HR: 1.16), while RFM levels below this threshold did not demonstrate a significant association with diabetes risk. A research project undertaken by Suthahar et al. (14) in the Netherlands observed a notable connection between RFM and the risk of new-onset type 2 diabetes, particularly among younger individuals. Cao et al. (18) investigated the link between RFM and non-alcoholic fatty liver disease (NAFLD), finding that RFM had a positive correlation with the possibility of NAFLD, with a threshold effect observed in both women and men, with thresholds of 34.95 and 23.40, respectively. Shen et al. (19) also found that RFM connected with the occurrence of NAFLD and cardiovascular disease in the Chinese population, emphasizing the potential of RFM as a disease prediction tool. Zwartkruis et al. (12) studied the relationship between RFM and atrial fibrillation, heart failure, and coronary heart disease, and the results showed that RFM was significantly associated with the occurrence of these cardiovascular diseases. Collectively, these findings underscore the notion that RFM serves not only as a reliable metric for evaluating body fat, but also potentially plays a substantial role in the prevention and control of diverse chronic diseases.

RFM showed a high correlation (*r* = 0.85 and *r* = 0.81) with percent body fat (BF%) and percent trunk fat (trunk fat%) measured by DXA (dual-energy X-ray absorptiometry) and BIA (bioelectrical impedance analysis) with a higher concordance (Kappa index) than BMI (20). Studies have shown that RFM more accurately identifies individuals with visceral obesity, and is particularly superior to BMI in distinguishing fat distribution and gender differences (21, 22). Visceral fat accumulation was strongly and positively associated with inflammatory markers (e.g., IL-6, leptin), and RFM was more significantly associated with these inflammatory markers than BMI (23, 24).

Visceral fat and body fat were significantly more strongly associated than BMI and waist circumference in assessing the risk of metabolism-associated fatty liver disease (25). In cardiovascular disease risk prediction, RFM was more strongly associated with abnormal glucose homeostasis, dyslipidemia, hypertension, and coronary heart disease, especially in men (26). The predictive power of RFM was superior to that of BMI in assessing both female infertility and gallstone risk (22, 27).

Furthermore, the association between various body fat indicators and arthritis has gradually attracted attention. Huang et al. (28) found in a cross-sectional research that there is a significant nonlinear relationship between lipid accumulation products (LAP) and OA. When LAP values are less than 120, the risk of OA increases significantly. Wang et al. (29) also employed NHANES data from 1999 to 2018 to examine the correlation between body roundness index (BRI) and the risk of OA. The findings indicated that for every unit increase in BRI, the risk of OA increased by 18% [OR = 1.18 (1.13–1.23), *p* < 0.0001]. In a related study, Yan et al. (30) examined the affiliation between the triglyceride-to-glucose index (TyG) and arthritis. The findings indicated that the TyG index showed a significant link with the likelihood of developing arthritis, particularly among adults of normal weight without diabetes (OR = 1.15, 95% CI: 1.07–1.23). In a related study, Wang et al. (31) examined the association between WWI and RA and OA. Their findings indicated a linear and positive correlation between WWI and OA, while the relationship with RA exhibited a complex nonlinear pattern with a significant threshold effect. According to a study by Wu et al. (32), high levels of fat-soluble vitamin A are associated with an increased risk of osteoarthritis. However, these studies focused only on the US population and were cross-sectional. In contrast, the present study was conducted in the Chinese and American populations and showed that RFM may be an independent risk factor for arthritis through the longitudinal study of the CHARLS group.

Obesity has been demonstrated to be a contributing factor to the development of arthritis, including OA and RA. The underlying mechanisms by which obesity exerts this influence are multifaceted and not yet fully elucidated. One hypothesis posits that the mechanical load hypothesis, which suggests that an increase in body mass leads to an elevated burden on joints that carry the loads, such as the knee and hip, resulting in cartilage degeneration and joint damage (33, 34).

The adipokines that are secreted by adipose tissue, which is an active endocrine organ, include leptin and adiponectin. These adipokines induce synovial inflammation and cartilage disintegration, which further exacerbates the degenerative alterations that are associated with OA (35, 36). The imbalance of adipokines secreted by adipose tissue in obesity, especially elevated leptin levels, activates inflammatory pathways such as IL-1β and NF-κB, promoting articular cartilage degradation and synovial inflammation, which is an important mechanism in the development of OA (37–39). The anti-inflammatory effects of lipocalin are well-documented; however, its role in OA is more complex. Research indicates that high levels of lipocalin may be associated with increased disease severity, suggesting a potential role in the exacerbation of inflammation (40, 41).

Elevated leptin/lipocalin ratios are theorized to be significantly associated with arthritic symptoms and cartilage destruction, and they have been identified as potential biomarkers of OA risk in obese populations (42, 43). A growing body of experimental and epidemiological evidence indicates that this elevated ratio induces enhanced catabolism inchondrocytes. Moreover, the antagonistic effect of lipocalin on leptin signaling is diminished in the context of obesity, thereby exacerbating the progression of OA (41, 44).

RFM reflects the accumulation of abdominal and visceral adiposity, and is closely related to adipokines released from visceral adipose tissue (VAT). Adipokines, such as leptin, secreted by VAT exert influence on the joint microenvironment through systemic circulation and local effects, including those on the knee fat pad (45–47). Research has demonstrated a negative correlation between VAT and lipocalin levels. Furthermore, increased VAT has been shown to be directly associated with elevated leptin concentrations in synovial fluid, which in turn accelerates cartilage degeneration (46, 48).

Furthermore, leptin levels are found to be more pronounced in female OA patients, and it has been posited that this may synergize with estrogen and BMI to explain the higher susceptibility to OA in women (37). Obesity-associated insulin resistance also synergizes with adipokines to promote metabolic disorders and inflammation in OA (49, 50). It is important to note that OA with elevated RFM is not solely caused by mechanical loading. OA in non-weight-bearing joints is similarly associated with obesity and adipokine imbalance, reflecting the systemic regulatory role of adipokines (48, 51, 52). Animal experimentation has demonstrated that leptin plays a pivotal role in the development of obesity-associated OA, though its function is restricted in the absence of an inflammatory background associated with obesity (39, 53, 54).

Leptin itself has been shown to have pro-inflammatory properties, which may contribute to joint inflammation by activating Th1 cells and pro-inflammatory cytokines (55, 56). Elevated leptin levels in obese individuals may also promote RA development through the activation of inflammatory pathways, such as JAK/STAT (57, 58). High molecular weight lipocalin isoforms may promote bone erosion (59).

The relationship between insulin resistance and arthritis is a subject of increasing research interest. The hyperglycemic state caused by insulin resistance is believed to exacerbate inflammation, damage cartilage cell function, and promote the formation of advanced glycation end products, creating an adverse microenvironment that further promotes the development of OA (60, 61). Low-grade systemic inflammation caused by gut flora imbalance is also considered an important factor in the development of arthritis. Increased intestinal permeability and the transfer of bacterial lipopolysaccharide (LPS) into the circulation have been demonstrated to trigger a systemic inflammatory response (62, 63).

This study boasts several notable advantages. First, it examined populations in both the United States and China. The study was conducted in the NHANES data set and verified in the CHARLS data set, enhancing the reliability of the results. Second, a cross-sectional study was complemented by a longitudinal study, providing substantial causal evidence for the association between RFM and arthritis. Notably, this study is pioneering in its exploration of the RFM-arthritis association, offering novel insights into the potential mechanisms underpinning this relationship.

Nonetheless, the present study is not without its limitations. First, although a longitudinal study was conducted, further evidence is needed to confirm the causal relationship between RFM and arthritis. In addition, due to the limitations of the research data, it is not possible to further determine the relationship between RFM and subtypes of arthritis. Finally, the potential for unaccounted confounding factors cannot be ruled out. To address these limitations, further evidence is needed to reveal the link between RFM and arthritis.

## Data Availability

The original contributions presented in the study are included in the article/Supplementary material, further inquiries can be directed to the corresponding author.
