# Supplementary material for: Association between relative fat mass and risk of arthritis: a study based on populations in China and the United States
Source: Front Nutr. 2025 Sep 2;12:1555135. doi: 10.3389/fnut.2025.1555135 (PMC12439532; doi:10.3389/fnut.2025.1555135)
Supplement: Supplementary file 1 [file Table_1.DOCX]

**CHARLS**

DA007 Have you been diagnosed with [conditions listed below, read one by one] by a doctor? 是否有医生曾经告诉过您有以下这些慢性病？

1. Hypertension 高血压病

2. Dyslipidemia (elevation of low density lipoprotein, triglycerides (TGs),and total cholesterol, or a low high density lipoprotein level) 血脂异常（高血脂或低血脂）

3. Diabetes or high blood sugar 糖尿病或血糖升高（包括糖耐量异常和空腹血糖升高）

4. Cancer or malignant tumor (excluding minor skin cancers) 癌症等恶性肿瘤（不包 括轻度皮肤癌）

5. Chronic lung diseases, such as chronic bronchitis , emphysema ( excluding tu mors, or cancer) 慢性肺部疾患如慢性支气管炎或肺气肿、肺心病（不包括肿瘤或癌）

6. Liver disease (except fatty liver, tumors, and cancer) 肝脏疾病（除脂肪肝、肿瘤或癌 外）

7. Heart attack, coronary heart disease, angina, congestive heart failure, or other heart problems 心脏病（如心肌梗塞、冠心病、心绞痛、充血性心力衰竭和其他心脏疾病）

8. Stroke 中风

9. Kidney disease (except for tumor or cancer) 肾脏疾病（不包括肿瘤或癌）

10. Stomach or other digestive disease (except for tumor or cancer) 胃部疾病或消化系 统疾病（不包括肿瘤或癌）

11. Emotional, nervous, or psychiatric problems 情感及精神方面问题

12. Memory-related disease 与记忆相关的疾病（如老年痴呆症、脑萎缩、帕金森症）

13. Arthritis or rheumatism 关节炎或风湿病

14. Asthma 哮喘

**NHANES**

Has a doctor or other health professional ever told {you/SP} that {you/s/he} . . .had arthritis (ar-thry-tis)?

|  |  |  |  |  |
| --- | --- | --- | --- | --- |
| **Code or Value** | **Value Description** | **Count** | **Cumulative** | **Skip to Item** |
| 1 | Yes |  |  |  |
| 2 | No |  |  |  |
| 7 | Refused |  |  |  |
| 9 | Don't know |  |  |  |
| . | Missing |  |  |  |
